# AIMAR survey on complex forms of bronchial asthma and COPD, their management and perception of critical issues

**DOI:** 10.1186/2049-6958-9-52

**Published:** 2014-10-28

**Authors:** Claudio F Donner, Alberto Visconti

**Affiliations:** AIMAR (Interdisciplinary Association for Research in Lung Disease), Mondo Medico, Borgomanero, NO Italy; AIMAR, ICT Consultant, Arona, NO Italy

**Keywords:** Bronchial asthma, COPD, Complex forms, Online survey

## Abstract

**Background:**

The management of patients with complex forms of bronchial asthma and COPD is not usually addressed in the major international guidelines and management documents which exclusively address pure forms. AIMAR thus undertook a survey to obtain information about: a) the perceived frequency of complex forms of asthma/COPD in adult patients and in the elderly; b) patient management regarding the complex forms (focus on therapeutic goals and consequent treatment); c) the management problems perceived in diagnosis, management, monitoring, indices of appropriateness in pharmacological treatment and adherence to treatment.

**Methods:**

The survey consisted of 18 multiple choice questions, completed by means of a web-based electronic form published in internet. All the data and responses inserted in the system were checked on-line for coherence and completeness directly during the phase of insertion and each participant had one only possibility of participating. The data thus collected were memorized directly within a relational database, based on consolidated open-source MySQL technology, and thus were immediately available for examination also during the course of the survey. Access to the data, mediated by a “back office” system of interrogation and report, enabled constant monitoring of the survey as it was being carried out, as well as extractions and verification, even on smaller data sets.

**Results:**

The survey was carried out in the full month of December 2013 and first half of January 2014. A total of 252 questionnaires were collected from the following physician groups: pneumologists (n = 180), general practitioners (GPs) (n = 32), allergologists (n = 8), internal medicine specialists (n = 20), other specialists (n = 12).

**Conclusions:**

Complex forms of bronchial asthma and COPD are frequently observed and considered present in variable percentages ranging from about 10% to about 50% of patients visited and considered typical of patients with a previous history of asthma. Risk factors such as smoking, obesity, bronchial hyperreactivity and genetic predisposition are considered important. Diagnosis is difficult solely on the basis of symptoms in approximately 50% of cases, and a previous history of asthma, history of spirometry and presence of allergy are of help. Treating inflammation and reducing exacerbations are considered the key therapeutic goals and the combination of inhaled corticosteroid (ICS) and long acting β_2_-agonist (LABA) and monotherapy with ICS are considered the fundamental pharmacological mode for treating patients with mixed forms of bronchial asthma and COPD. Treating with only a bronchodilator is considered to be moderately risky for this type of patient. The identification and management of mixed forms result more impeded by “logistic” aspects, e.g. long waiting lists and integration with the GP, than by aspects intrinsic to the disease management itself, e.g. selecting the assessment or interpreting the outcome of the instrumental examinations. Treatment continuity and the integration between GP and specialist are the factors that most limit the management of mixed forms in the stable phase.

## Background

Bronchial asthma and chronic obstructive pulmonary disease (COPD) are chronic diseases that are very widespread and are characterized by an increasing epidemiological trend. It is estimated that asthma affects about 4% of the adult population and 9-13% of the pediatric population in developed countries, including Italy. COPD affects approximately 5% of the general population, is concentrated in the adult and elderly age-ranges, and the prevalence can reach rates of even over 20% in the subgroup of males aged over 60 years [1].

Both diseases are characterized by the presence, with diverse features, of airways inflammation and airways obstruction, with respiratory symptoms that are often in part identical. They are, however, two clearly distinct diseases, with specific pathophysiological and clinical characteristics that sharply differentiate them. But cases are not infrequent of single patients, particularly amongst the elderly, in whom it is difficult to make a differential diagnosis due to “complex” syndromes where asthma and COPD overlap in the same patient, such as can occur, for example, in an asthmatic individual exposed to inhalation of harmful substances, like cigarette smoke. The diagnosis and management of such patients are unfortunately not dealt with in the major international guidelines - such as GOLD for COPD and GINA for asthma - that refer to pure forms, completely excluding the complex forms.

Hence there is clearly an interest in investigating how physicians who habitually follow patients with obstructive airways disease perceive the impact of the “complex ”forms of bronchial asthma/COPD in their clinical practice, so as to evaluate their real role in clinical practice and be able to provide in future reliable indications on the most suitable modes of identifying these forms as well as on their optimal management. On this basis AIMAR undertook to carry out, with the support of an unrestricted educational grant from TEVA, a survey on this specific topic, aimed at obtaining information on the following aspects:

- Perception of the frequency of complex forms of asthma/COPD in adult and elderly patients;
- mode of clinical management of patients with forms characterized by the presence of both components, with particular regard to the therapeutic goals and consequent treatment;
- perception of possible difficulties of management in the diagnosis, treatment, monitoring, and evaluation of the indices of appropriateness in pharmacological treatment and of adherence to treatment.

## Methods

A working group of qualified experts elaborated, on the basis of the above goals, a survey designed to acquire data through a dynamic “form”, always available in internet, able to be completed at any moment by the person concerned, in a few minutes and with a few simple interventions. The categories of specialists to whom the survey was administered were basically three (pneumologists, allergologists, and internal medicine specialists) with extension to general practitioners (GPs). The survey consisted of 18 multiple-choice questions completed by means of a web-based electronic form published in internet - time required for completion of the survey was not more than 15 minutes. All the data and responses inserted in the system were checked on-line for coherence and completeness directly during the phase of insertion and each participant had one only possibility of participating. These measures ensured verification of the data at the survey was being conducted, eliminating cases of partial data and possible duplications due to multiple participation (which would obviously have lowered the significance of the final results).

The data thus collected were memorized directly in a relational database (based on the consolidated open-source technology MySQL, a *de facto* standard in web applications) and thus were immediately available to examination also during the period while the survey was being conducted. Access to the data, mediated by a “back office” system of interrogation and report, enabled constant monitoring of the survey as it was being carried out, as well as extractions and verification, even on smaller data sets.

The survey was carried out during the full month of December 2013 and first half of January 2014: in this period a total of 252 physicians responded to the survey; they were distributed among the above-mentioned categories that took part, thus enabling a heterogeneous and interdisciplinary overview of the topic under investigation. The enrollment of participants highlighted the positive synergy between “web-based” technologies [2] and the social networks [2, 3] alongside the normal, classic system of invitation to participate carried out by bulk mailing. The initiative was promoted at several occasions through the social network channels where AIMAR has for some time been present. Through its own page in Facebook (institutional group profile [2] and official Twitter channel [3]), it was possible to reach rapidly a large number of heterogeneous participants spread throughout the national territory (confirming once again the “viral” effect of the social networks in involving people and circulating information).

## Results and discussion

Overall 252 questionnaires were collected, subdivided into: Pneumologists = 180,GPs = 32, Internal Medicine specialists = 20, Allergologists = 8, other Specialists = 12.

### Epidemiological aspects

**Figure 1:**
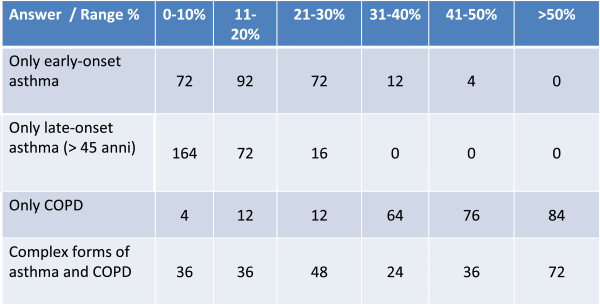
**Considering your patients with bronchial asthma and/or COPD, on average what percentage have.**

In the sample of physicians interviewed (Figure 1), complex forms of asthma and COPD were retained to be frequently encountered and present in a percentage varying from approximately 10% to 50% of patients visited; this is substantially in line with evidence from retrospective studies that report estimates of prevalence up to approximately 28.5% [4–6]. The remaining part of respiratory patients was composed of individuals with COPD only, individuals with asthma only with childhood onset, and individuals with asthma only with late onset (over 45 years).

Complex forms of bronchial asthma and COPD are retained typical of patients with a previous history of asthma. It is now known that forms or phenotypes of severe chronic bronchial asthma exist and have similar characteristics to those of COPD, namely: accelerated lung function decline with a progression towards poorly reversible bronchial obstruction, neutrophils in sputum and/or inflammation difficult to control by therapy, elevated level of bronchial hyperreactivity, and frequent exacerbations [4, 7–10].

**Figure 2:**
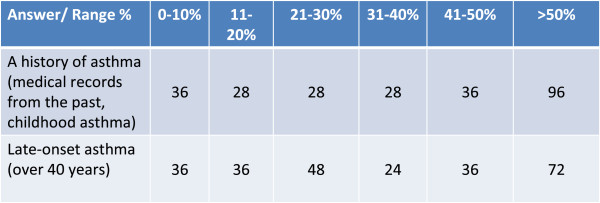
**Among the patients with complex forms of overlapping asthma and COPD, on average how many, as a percentage, have.**

**Figure 3:**
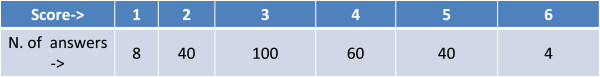
**On average, in patients aged > 40 years, do you have difficulty distinguishing late-onset asthma from COPD and complex forms based on symptoms alone? (Score: 1 = never…6 = always).**

These forms (also known as “complex” forms of asthma and COPD) are very frequent in asthmatic smoker patients, who typically tend to evolve towards a clinical picture resembling COPD both functionally (bronchial obstruction increasingly less reversible) and pathologically, developing a mixed form of asthma- and COPD-like inflammation, characterized by the concomitant presence of eosinophils, CD8 lymphocytes and neutrophils [4].Besides the so-called “complex” forms, another factor than can hinder the differential diagnosis between the two diseases is the fact that asthma can also have a late onset, becoming an important cause of dyspnea and functional limitation in the elderly. These forms of asthma with late onset are often misdiagnosed or interpreted as COPD (in only 1 case out of 5 a history of allergy is found) and appear to have (Figure 2) a considerable impact in clinical practice posing problems for the diagnosis (Figure 3).

### Diagnosis

**Figure 4:**
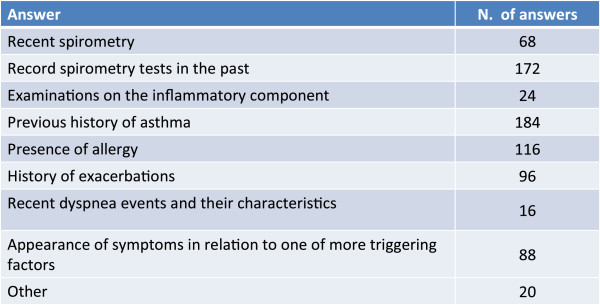
**Besides symptoms, what other elements mostly suggest to you that your patient may have a complex form of overlapping asthma and COPD?**

In the majority of cases it results difficult to diagnose the complex forms based just on symptoms and a previous history of asthma - spirometry assessments over the course of time and the presence of allergy are considered important in the diagnostic phase (Figure 4).

The assessment solely of respiratory symptoms (dyspnea, cough, sensation of chest constriction) as well as a single spirometry test do not generally permit to recognize a form in which bronchial asthma and COPD overlap. In fact, as reported above, there are cases of asthma (of long duration, in smokers or the elderly) with poor or even absent reversibility, and, viceversa, cases of COPD with a certain amount, even significant, of reversibility, perhaps due to the coexistence of an asthmatic component.

The diagnostic establishment of “complex” forms of asthma and COPD is thus difficult and must take into account the overall assessment of the patient based on an in-depth clinical-anamnestic examination: type and mode of onset of symptoms (association to triggering factors), previous history of atopy or asthma, history of smoking, spirometry data with reversibility, previous episodes of bronchial hyperresponsiveness, clinical signs and x-ray findings.

### Risk factors

**Figure 5:**
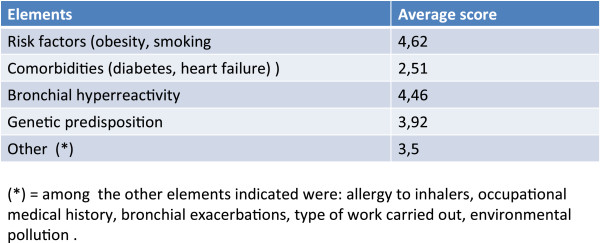
**On a scale from 1 to 6 what importance do the following elements have in the onset of complex forms with overlapping asthma and COPD?**

Risk factors such as smoking, obesity, bronchial hyperreactivity and genetic predisposition are considered important (Figure 5). As stated earlier, exposure to smoking (or occupational exposure to air pollutants) in asthmatic patients is considered to be the principal risk factor for evolution towards a clinical picture indicative of COPD (or for the development of mixed or overlapping forms). It should also be emphasized that smoking reduces the efficacy of steroid anti-inflammatory therapy [4], aggravating the risk of exacerbations. Also obesity seems to be a risk factor for difficult-to-control asthma (presumably due to the activation of common pro-inflammatory mechanisms), with possible reduced response to corticosteroids and accelerated decline of lung function [10]. Concerning the role of airway hyperreactivity, typically present in asthmatic patients and demonstrable in almost 1/3 of patients with COPD, some studies suggest that it can constitute a risk factor for a more rapid evolution of bronchial obstruction [4, 11].

It should be recalled that also a poorly developed lung at birth, due to nutritional deficits or toxic-infective factors occurring *in utero* or in the early phases of life, can render the respiratory system more vulnerable to external agents (e.g. smoking, viral infections) and represent a risk factor for the early development of an obstructive condition that is poorly reversible [4, 12].

Finally, both asthma and COPD can be considered the result of a complex interaction of endogenous and exogenous factors. External agents (allergens in asthma and smoking in COPD) presumably act on a terrain that is constitutionally susceptible (genetic predisposition) creating favorable conditions for the initiation of inflammatory and obstructive processes.

### Treatment goals

**Figure 6:**
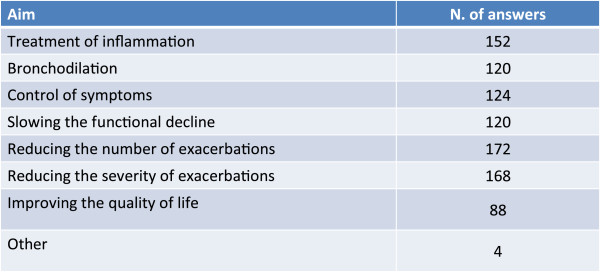
**In patients with complex forms of overlapping asthma and COPD, the main treatment goals are.**

Reduction of the number and severity of exacerbations and the treatment of inflammation are considered to be the main goals of therapy (Figure 6). In both bronchial asthma and COPD the pathogenically determining element consists of a chronic inflammation of the airways (even if with diverse cell mediator characteristics) that can progressively lead to structural modifications, worsen the obstructive picture and increase the risk of exacerbations (events that profoundly impair the quality of life of the patient and are the principal source of health resources consumption in chronic obstructive bronchial diseases). On this basis, the sample of physicians that took part in the survey retained that treatment of the underlying inflammation and the reduction of exacerbations and of their severity were the principal goals of treatment of the complex forms.

It is well known that in eosinophilic asthma the response of the inflammatory and clinical picture to inhaled steroid therapy is excellent: reduction of the cell count, improvement of bronchial hyperreactivity, prevention of exacerbations and improvement of quality of life [10]. However, the recent study GLUCOLD carried out in patients with COPD, excluding those with a current or past history of bronchial asthma, found that long-term treatment with inhaled steroid significantly reduced non eosinophilic bronchial inflammation (mast cells, CD8+ and CD3 + lymphocytes), increased the epithelial integrity and improved the clinical picture [13]. The addition of a beta2- stimulant improved the spirometry indices and dyspnea, even if it did not have an effect on the bronchial inflammation. The specificity of the effects of the steroid has been demonstrated by the observation that its suspension determined a resumption of the inflammatory process and a worsening of symptoms and of spirometry parameters [13].

### Therapeutic approach

**Figure 7:**
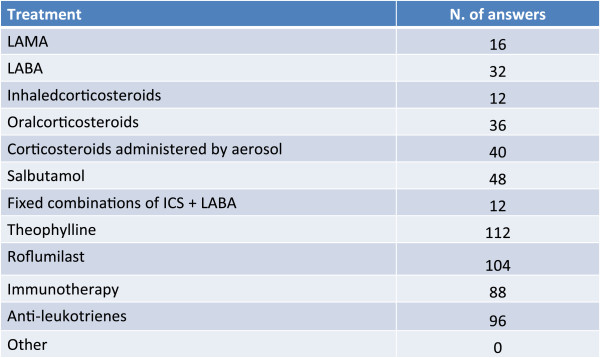
**What treatments do you exclude in complex forms of overlapping asthma and COPD?**

**Figure 8:**
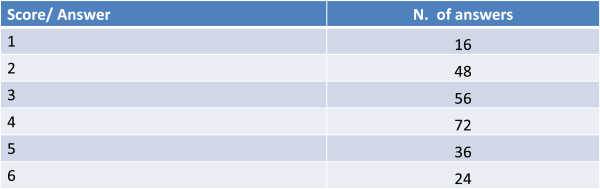
**The FDA recently cautioned against the sole use of LABAs in asthmatic patients, so as to avoid masking the symptom and a concomitant worsening of the disease.** In your opinion, how “risky” is the sole use of a bronchodilator for a patient with mixed forms?

Inhaled corticosteroids (ICS) variously associated to bronchodilators constitutes a pharmacological strategy that is considered fundamental for the treatment of patients with complex forms of bronchial asthma and COPD, while phosphodiesterase inhibitors such as theophylline and roflumilast seem to play a secondary role (Figure 7).Treatment solely with the bronchodilator is considered on average at risk of masking the disease in this type of patient (Figure 8).

**Figure 9:**
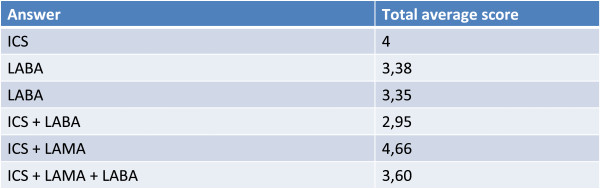
**Considering that both asthma and COPD are chronic diseases, in the case of complex forms how important do you consider treatment continuity to be with the following treatment options?**

Continuity of treatment results more important for ICS and combination drugs, such as ICS + a long-acting β_2_-agonist (LABA), than for the bronchodilator used alone (Figure 9): this could reflect a use of bronchodilators (LABA or long-acting muscarinic antagonists [LAMA]) either in less severe patients or tendentially as needed.

The fundamental role of treatment continuity with ICS regimes for attaining the clinical outcomes, both in bronchial asthma and in COPD, is supported by multiple evidence in the literature [14–17]. For example, the COSMIC study has shown that the suspension of inhaled corticosteroid in patients with moderate-severe COPD stabilized with the combined therapy fluticasone/salmeterol led to a rapid and significant deterioration of respiratory function and symptoms, confirming the key role played by this class of drugs as maintenance therapy for COPD [16].

In line with the opinion that overlapping forms can have an asthmatic origin (or are forms of late onset asthma) and that treating the inflammation together with reducing exacerbations are the principal goals of pharmacological therapy, continuing treatment with ICS, both alone and in association with a LABA, is considered fundamental for the treatment of these patients. However, in contrast with these observations, from the survey it emerged that many physicians do not consider it right to exclude the use of LAMAs or LABAs in monotherapy, considering the use of the bronchodilator alone (without inhaled corticosteroid) only “moderately at risk” (Figure 8). On this point, it should be emphasized that LAMAs are currently indicated only for the treatment of COPD and that the use of LABAs in asthma is subject to precaution: the U.S. Food and Drug Administration has in fact recommended that in asthma LABAs (salmeterol or formoterol) should always be prescribed as additional therapy to inhaled corticosteroids, when the latter alone do not provide an adequate control of symptoms, specifying moreover that, if appropriate, use of a combined LABA-corticosteroid product for inhalation may be preferable to the single components, as means to increasing the compliance to the prescribed treatment [18].Concerning treatment of the overlapping forms it is important to recall that the major international guidelines address the pure forms (GINA for bronchial asthma and GOLD for COPD). The therapeutic goals of GINA and GOLD have many elements in common (relief of symptoms, prevention and treatment of exacerbations, improvement of the quality of life, good tolerability of the pharmacologic treatments, etc.), but the therapeutic approach is in part reflective (Figure 9): in asthma ICS constitute the driver of the therapy, to which LABA bronchodilators are added to achieve/improve control in patients who are symptomatic with ICS alone, whereas in COPD bronchodilators represent the first step of treatment, to which ICS can be added when indicated (in patients with frequent exacerbations, etc.) in the severe to very severe forms. In forms where asthma and COPD overlap, the combined use of ICS and LABA can be appropriate.

Of the physicians who responded to the survey, most (184 out of 252) retain that there is no standardized pharmacological treatment for COPD patients and that thus this has to be tailored to the individual patient. The net minority (68 out of 252) who retain that there exists a standardized treatment for COPD patients consider prevalently bronchodilators, used either singly (20) or an associated use of bronchodilators (8), as the primary approach as opposed to the association of ICS + bronchodilator (12) or other interventions ( 24) such as, for example, LAMAs or roflumilast.

### Critical problems in management

**Figure 10:**
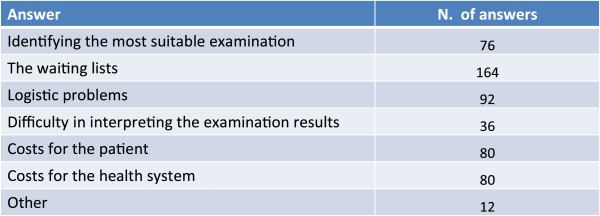
**Which are the main obstacles in using the diagnostic tools?**

**Figure 11:**
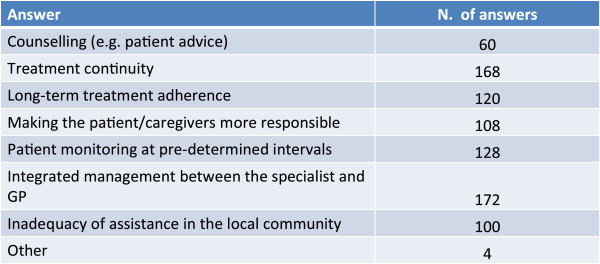
**In relation to management of the stable phase of the disease between hospital and community health-services, which are the main difficulties that you encounter?**

The recognition and the management of mixed forms are hindered more by difficulties of a technical-administrative nature such as the long waiting lists, logistic difficulties and costs, than by aspects strictly inherent to the disease management itself, such as selecting the most suitable examination and interpreting the assessment results (Figure 10).Treatment continuity and the integration between GP and specialists are the factors that most limit the management of complex forms in the stable phase, followed by monitoring over time of the patient and the long-term adherence to treatment (Figure 11).

**Figure 12:**
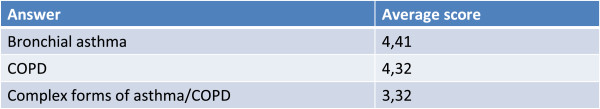
**What importance do you ascribe to the current guidelines in the following cases?**

The international guidelines (GINA for asthma and GOLD for COPD) deal with pure forms, excluding patients in whom there is an overlap of the two diseases from the specific management recommendations. In fact, in the GINA guidelines COPD is indicated as a comorbidity of asthma, along with other diseases such as rhinitis and gastroesophageal reflux disease [10], while in the GOLD guidelines asthma is indicated as a risk factor for the development of COPD [1]: in neither of the two documents, however, there are references to the therapeutic management. It should also be highlighted that patients with “complex” forms are often excluded from clinical studies for only asthmatic or only COPD patients, in that their etiopathological, clinical and functional characteristics fail to satisfy the inclusion criteria. It thus emerges the importance of carrying out trials designed specifically for this patient population, with distinct clinical outcomes, and the need to formulate specific recommendations for the management of the overlapping forms (Figure 12).

People with an overlap of asthma and COPD, for whom the term “asthma-like bronchitis” was once used, present an inflammatory and obstructive picture that can benefit from early use of the combination of inhaled corticosteroids and long-acting bronchodilators, such as LABAs.

**Figure 13:**
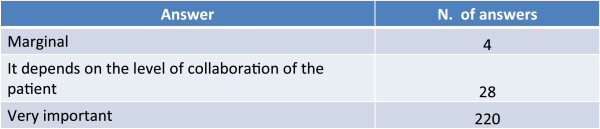
**What role do you attribute to quality and ease-of-use of the device in achieving the treatment goals?**

**Figure 14:**
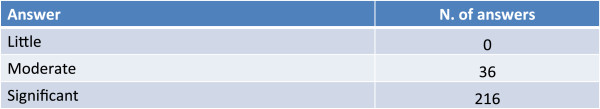
**What influence in your opinion do the features of the device (quality, ease-of-use, etc.) have on long-term adherence to treatment?**

As with all chronic diseases, for complex forms as well as for the pure forms of bronchial asthma and COPD patient’s adherence to treatment is essential.One of the elements that can influence adherence is the choice of the inhaler device. The vast majority of physicians who responded to the survey retain the quality and ease of use of the device highly important to achieve both the therapeutic goals (Figure 13) and a good adherence to treatment (Figure 14). Of interest to note, physicians declared they dedicate a rather long time (on average 8.67 min) to educating the patient when they prescribe a drug administered by inhalation; this contrasts with the widespread notion that doctors are not very sensitive to these aspects.

## Conclusions

The major elements that emerged from the survey are the following:

- The physicians involved in the survey were in general very conservative and prudent in their replies and we had the clear feeling that they were afraid not to give correct or orthodox replies to some critical questions.
- Complex forms of bronchial asthma and COPD are considered, by the sample of physicians who took part in the survey, to be present in a variable percentage ranging from about 10% to about 50% of patients visited.
- Complex forms are considered to be typical of patients with a previous history of asthma, particularly if smokers.
- Risk factors such as smoking, obesity, bronchial hyperreactivity and genetic predisposition are considered to be important for the onset of a complex form of asthma and COPD.
- In the majority of cases complex forms of asthma and COPD are difficult to diagnose based on symptoms alone. A previous history of asthma, spirometry records and the presence of allergy are held to be important for the diagnosis.
- Reducing the frequency and severity of exacerbations and treating the inflammation are considered to be the principal therapeutic goals for the treatment of the complex forms of bronchial asthma and COPD.
- Inhaled corticosteroids (ICS) variously associated to bronchodilators constitute a pharmacological presidium considered fundamental for the treatment of patients with complex forms of bronchial asthma and COPD. Treatment with bronchodilators alone is considered on average at risk of masking the symptoms in this type of patient.
- The recognition and management of complex forms is hampered more by technical-administrative difficulties, such as long waiting lists, logistical problems and costs, than by aspects inherent to the management of the disease such as the identification of the most suitable examination and the interpretation of the results of instrumental examinations.
- Treatment continuity and the integration between the GP and the specialist are the factors that most limit the management of complex forms of asthma and COPD in the stable phase.
- The current guidelines for bronchial asthma and COPD are considered important for the management of the “pure” pathologic forms but less so for the complex forms: in neither document, in fact, is there reference to the diagnosis and management of this type of patient.
- The quality and the ease of use of the device are highly important for achieving the therapeutic goals and adherence to therapy.
